# Calibration of In-Plane Center Alignment Errors in the Installation of a Circular Slide with Machine-Vision Sensor and a Reflective Marker

**DOI:** 10.3390/s20205916

**Published:** 2020-10-20

**Authors:** Hyungjin Jeong, Jiwon Yu, Donghun Lee

**Affiliations:** School of Mechanical Engineering, Soongsil University, Seoul 06978, Korea; hyungjinjeong2@soongsil.ac.kr (H.J.); jiwon1123@soongsil.ac.kr (J.Y.)

**Keywords:** machine vision, in-plane center-alignment error calibration, circular slide, circle fitting, parallel mechanism

## Abstract

This paper describes a method for calibrating in-plane center alignment error (IPCA) that occurs when installing the circular motion slide (CMS). In this study, by combini ng the moving carriage of the CMS and the planar PKM (parallel kinematic mechanism) with the machine tool, the small workspace of the PKM is expanded, and the workpiece is placed on the table with the CMS installed is processed through the machine tool. However, to rigidly mount the CMS on the table, the preload between the guide and the support bearings must be adjusted with the eccentric bearing, and in this process, the IPCA occurs. After installing a reflective marker on the PKM, the PKM is slowly rotated along with the ring guide in the way of stop-and-go without the PKM’s own motion. Then, using a machine vision camera installed at the top of the CMS, the IPCA, which is the difference between the actual center position and the nominal center position of the CMS with respect to the camera, can be successfully calibrated through the circular fitting process. Consequently, it was confirmed that the IPCA of 0.37 mm can be successfully identified with the proposed method.

## 1. Introduction

The structure of the manipulator used as industrial robots can be mainly divided into a serial kinematic mechanism (SKM) and a parallel kinematic mechanism (PKM). The PKM is naturally associated with a set of constraint functions characterized by its kinematical closure constraints. The PKM has a closed-loop mechanical structure composed of two or more mutually connected links and has relatively high structural stiffness compared with the open chained structure. However, due to its structural characteristics that are mutually constrained, various types of singularities exist inside of its workspace [1,2], as well as the workspace is very small. To overcome these limitations from its inherent structural characteristics of the PKM, various studies have been conducted to eliminate the actuator and end-effector singularities, as well as to efficiently enlarge the usable workspace [3]. F.C. Park and J. W. Kim [4] used differential geometric tools to study PKM’s singularities and provided a finer classification of singularities. In their later works, they proposed the way of actuator redundancy as a means of eliminating actuator singularities and enlarging the usable spatial workspace. Consequently, a six-axis spatial PKM was manufactured based on this principle [5,6]. Gao. F described the characteristic of the workspace according to the variable link length and studied the workspace optimization of the parallel instrument, and Merlet, J.P. studied 3D or 6D boxes obtained from an introductory approach into this area. In addition, Ryu, S. J. carried out workspace optimization by adding linear joint design, or motion guide, to the instrument. Many studies have been conducted, such as performing the optimal design of the device itself, or installing the device on top of the motion guide [7,8,9,10,11,12,13,14,15].

**Figure 1:**
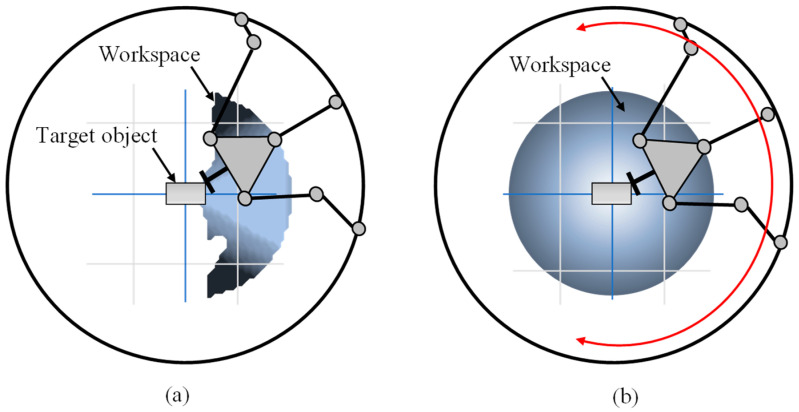
(**a**) Workspace of the fixed parallel kinematic machine, (**b**) workspace of parallel kinematic machine sweeping the center axis.

Due to the nature of the PKM for machine tools in this study, the end effector, as shown in Figure 1, should be capable of approaching and performing operations in any direction of the workpieces. Thus, by combining the PKM with machine tools with the moving carriage on the ring guide, the workspace was expanded by rotating the singularity-free workspace of the PKM about a vertical axis passing through the center of the ring guide.

However, ring slides, together with linear slides, have been used extensively as transfer systems for machine tool and automation applications [16].

**Figure 2:**
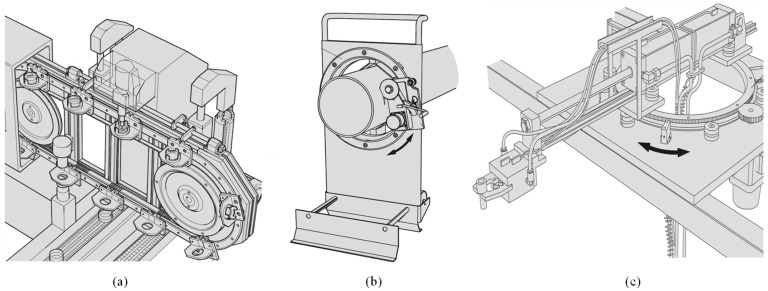
Application of circular and linear slide: (**a**) Optical lens assembly machine, (**b**) moving saw for tube cutting, (**c**) pick and place robot.

Figure 2 shows the applications that perform the process through ring slides and linear slides. (a) Optical lens assembly machines are the most representative form of industrial processes using motion guides, and ring slides are used for directional switching purposes and carry out transport operations through linear slides. (b) The moving saw for tube cutting is a piece of equipment made to cut the tube, and a cutting saw placed on the ring slide moves along the ring slide and cuts the long tube. (c) The pick and place device, with a ring slide equipped with a series instrument performing a steady 360° rotational motion. As such, ring slides have been mainly used for the purpose of turning the transportation route and have been used in areas where precision is not required much even if the process takes place on the ring slide.

**Figure 3:**
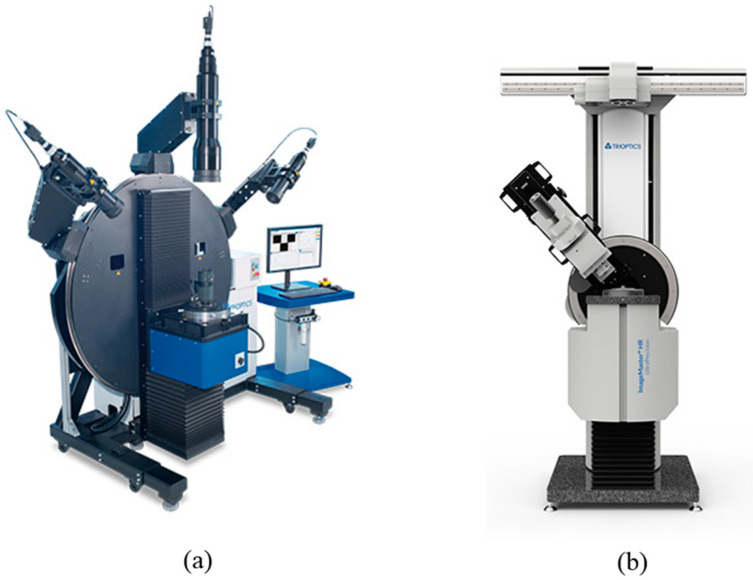
Tripotics Products: (**a**) Image master cineflex (**b**) Image master HR/universal.

However, like Figure 3, a variety of applications, including Trioptics high precision optical measurement system ‘Image master crane flex’, a measurement camera was mounted on the ring slides to ensure 10 um of Flange focal length acuity and ±0.2% effective local length accuracy [17]. This shows that ring slides can be used in applications that require relatively precise control.

However, there is an important difference between this study and Trioptics’ application of ring slides, in ‘Image master cineflex’ has a structure in which the ring slide is fixed on the platform on which the ring slide is based, and the bearing and pinion with a measurement camera on it rotates. In this study, on the other hand, the bearings, and motors secure the rotating pinion to the platform as the base, above which the PKM-equipped ring slide rotates.

**Figure 4:**
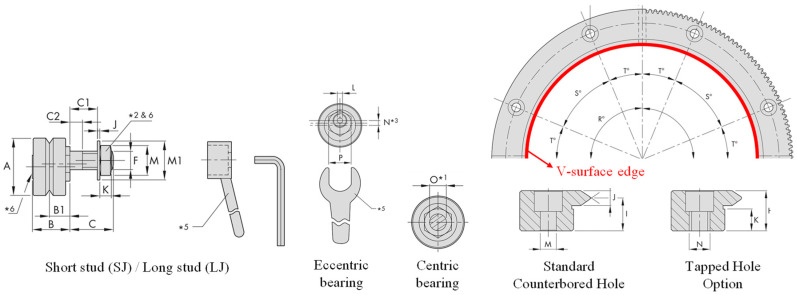
V-bearings of the single-edge ring system and circular motion slide system.

Figure 4 shows the V-bearing and circular motion slides of an external single-edge ring system. When rigidly integrating the V-surface edge of the ring slide with three support bearings, two concentric bearings, and one eccentric bearing should be evenly spaced at 120° intervals according to the installation guide. In the case of the eccentric bearing used in this study, the eccentric offset between the central axis of rotation and the stud axis of the bearing is 1.9–5.5 mm. The preload adjustment using this eccentric offset can prevent improper slide operation owing to positional dimension errors between the bearing mounting stud holes of the base.

**Figure 5:**
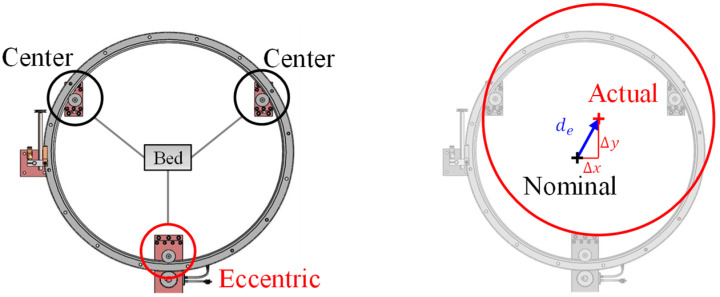
The center alignment error is caused by the eccentric bearing of the circular slide.

However, as shown in Figure 5, a difference between the nominal and actual dimensions of the center of the slide against the base reference occurs. In this study, the process is carried out by rotating the ring slide directly, so the in-plane center alignment error (IPCA) that can occur when installing the ring slide must be identified and calibrated because it seriously affects the relative positioning accuracy between the workpiece fixed inside the ring slide and the cutting part rotating along the circular guide [18,19,20]. 

Thus, in this study, a machine vision camera-based calibration method is proposed to identify this IPCA with a reflective marker mounted on the T-shaped fixture at the end-effector of the PKM. The retro-reflective marker-based real-time positioning and localization with the machine vision camera has been actively used in fields requiring precise measurement, such as robotic neurosurgery [21,22], until recently. After installing a reflective marker on the PKM, the PKM is slowly rotated along the ring guide in the way of stop-and-go without the PKM’s own motion, and then the automated marker localization process is performed to obtain the position of the marker with respect to the camera installed on the top of the circular motion slide (CMS). After all the center coordinates of the marker with reference to the camera coordinate system were obtained through this look-then-move process, the centroid coordinates of the CMS are estimated with the circular fit of the set of reflective-marker’s positions. As a result, the IPCA can be determined by calculating the relative distance from the nominal origin.

## 2. Theoretical Background

### 2.1. Definition of IPCA in CMS

Premise 1: The origin of all the coordinate systems to be described later is coplanar.

Premise 2: Frame {C} and frame {E} estimated through circle fitting have the same orientation.

(1) REC≡I

Premise 3: The nominal reference frame {$N_{r}$} and the nominal center frame {$N_{c}$} of the circular slide have the same orientation.

(2) RNcNr≡I

**Figure 6:**
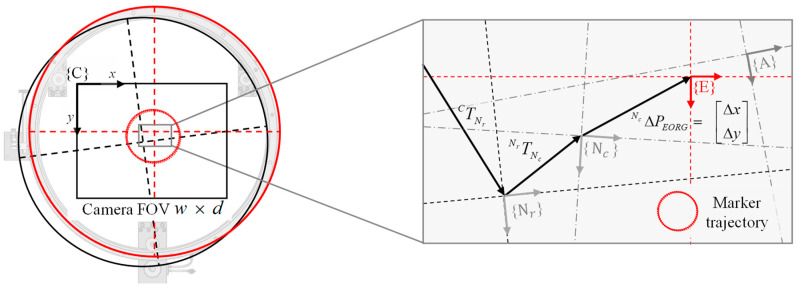
Schematic of the in-plane center alignment error (IPCA) of the circular motion slide (CMS), due to the uncertain assembly of the eccentric typed bearing.

Figure 6 shows the camera frame {C}, reference nominal frame {*N_r_*}, nominal center frame of the “design” slide {*N_c_*}, actual frame {A}, tool frame {T} attached to the T-shaped calibration tool, and frame {E} estimated according to the trajectory of the frame {T} and the field of view (FOV) of the camera. Because the actual location {A} of the CMS center is unknown, to define the nominal origin, a reference square is installed with three reflective markers attached to the “design” center on the optical table where the mechanism and camera are mounted, and {*N_c_*}, is defined. The detailed method of defining a nominal center frame is described in Chapter 3.

In this study, we aim to identify the alignment errors *^Nc^* ΔP*_E_._ORG_* between the origins of two coordinate systems through frame {*N_c_*} and frame {E} expressed with reference to frame {C}, as indicated by Equations (3) and (4).

(3) TNrC⋅TNcNr=TNcC

(4) ΔNcPE.ORG=ΔCPE−ΔCPNc

To estimate {E}, a T-shaped tool with a reflective marker attached to the edge of the cutting part is installed, the marker moves on the cutting part at a constant angular displacement along the CMS (counterclockwise, CCW), and “look-then-move” [23] is repeated, where the camera recognizes the position of the marker with reference to {C}. Five images are captured at each position, and the mean position of the marker is estimated through the machine-vision process with reference to frame {C}. Through this process, with circle fitting of the collected trajectory of the marker, the origin position of the coordinate system {E} is obtained. In this case, all the measurement coordinates are expressed with reference to frame {C}.

**Figure 7:**
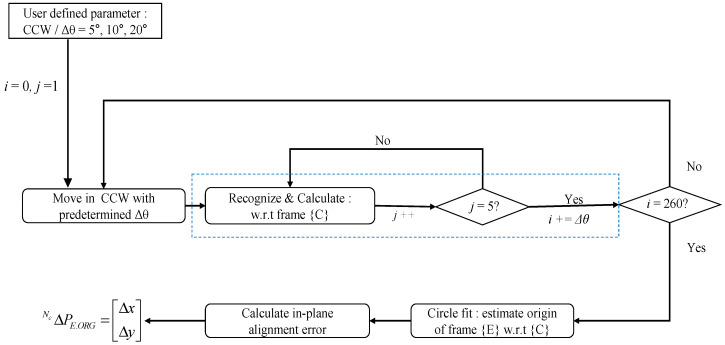
Flowchart of the look-then-move machine-vision process.

The entire process of look-then-move is summarized in Figure 7.

### 2.2. Measurement System Configuration

**Figure 8:**
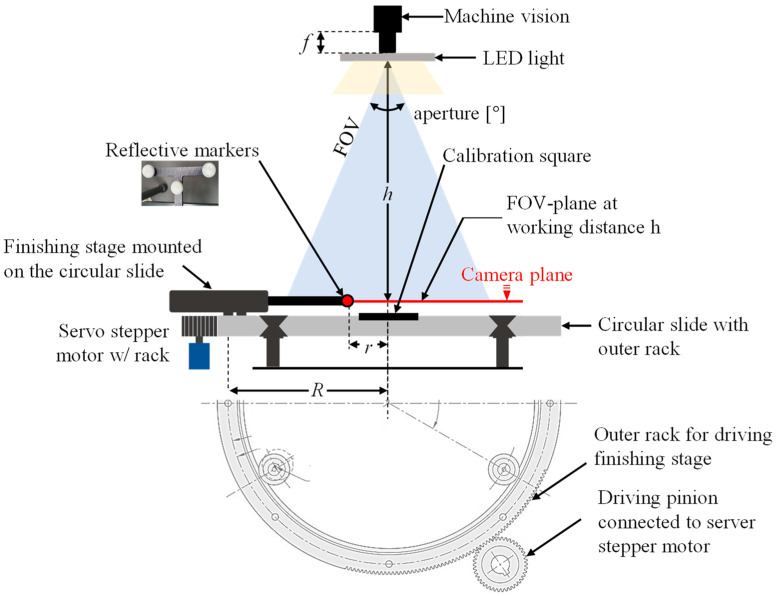
Schematic of the entire measurement system.

As shown in Figure 8 and Table 1, the measurement system consists of a machine-vision camera, dimmable light-emitting diode (LED) lights, a CMS, a parallel cutting part, a calibration tool, reflective markers, a reference square, and a controller.

(5) w=(n×h)/f

(6) d=(t×h)/f

(7) $$\left(\frac{f\times \alpha }{n}\right)\cdot s=\left(\frac{f\times \beta }{t}\right)\cdot s=h$$

(8) Resolution=w/α=d/β=s

Here, *h* represents the height from the calibration tool to the camera, *R* represents the radius of the CMS, *r* represents the radius of rotation of the marker, *w* represents the width of the FOV at *h*, and *d* represents the depth of the FOV at *h*. The main parameters related to the camera and lens selection are the working distance *h*, which is the installation height of the camera; the minimum FOV width *w* of the camera; the depth *d*; the sensor width *n*; the depth *t*; and the pixel size *m* of the image acquired by the camera. Additionally, *f* represents the focal length between the camera and the lens, and α and β represent the horizontal and vertical pixel resolutions of the camera, respectively. The factor that determines the measurement precision of the entire measurement system is the size of the unit pixel (in the unit of mm), and this value is determined linearly according to the working distance *h* and the installation height of the camera, after the machine-vision camera is selected. Therefore, in this study, the camera and the working distance were determined using Equations (5)–(8) so that the minimum size (in mm) of the unit pixel would be approximately 0.05 mm. The results are presented in Table 2. The detailed specifications of the camera and lens were presented in a previous study [24].

**Figure 9:**
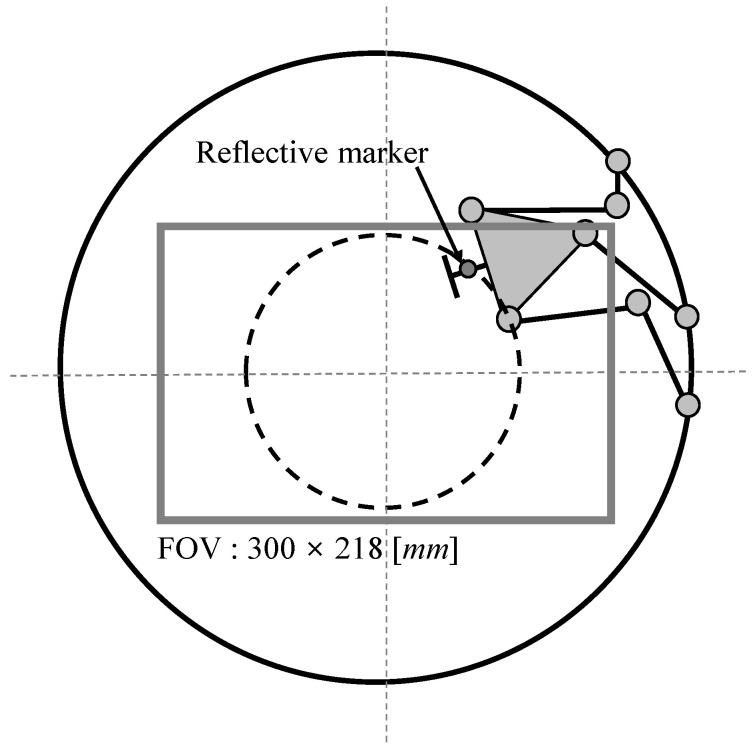
Schematic of the FOV and marker position.

At this time, as the calibration tool that functions as the end effector of the mechanism is closer to the CMS that serves as a supporting base, the vibration resulting from the position transition of the look-then-move process is minimized. As shown in Figure 9, the look-then-move experiment was performed upon adjusting the circular trajectory to be circumscribed to the greatest possible extent within the given FOV.

## 3. Experiment and Analysis

### 3.1. Calibration of Camera

**Figure 10:**
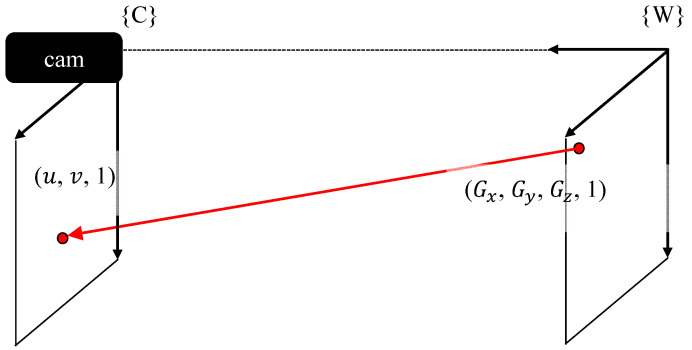
Mapping world coordinates to pixel coordinates.

Prior to calibrating the IPCA using the previously selected machine-vision camera, as shown in Figure 10, camera calibration should be performed. Here, error factors are identified and calibrated, such as the distortion of the lens (including the radial distortion and tangential distortion that arise when a point in a three-dimensional space is mapped onto a two-dimensional image plane) and installation uncertainties [25,26,27].

The camera calibration error factors are mainly divided into external and internal factors. The external factors include the working distance of the camera, the light intensity, and the horizontal accuracy of the camera, and the internal factors include the focal length f (the distance between the lens center and the image sensor) and the principal point (the image coordinate of the foot of the perpendicular from the center of the lens to the image sensor).

**Figure 11:**
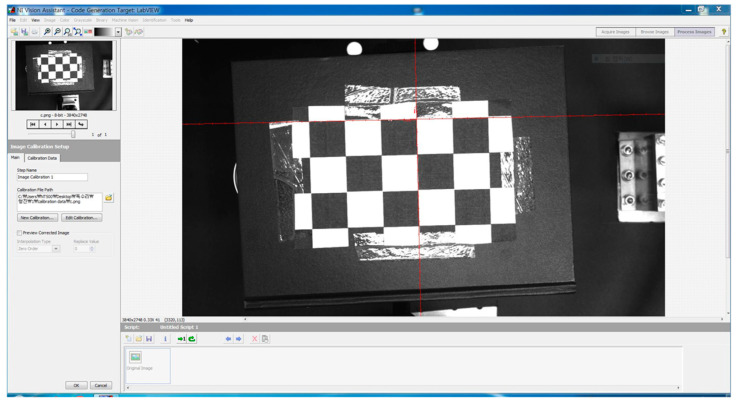
Snapshot of the NI Vision Assistant-based machine-vision camera calibration.

**Figure 12:**
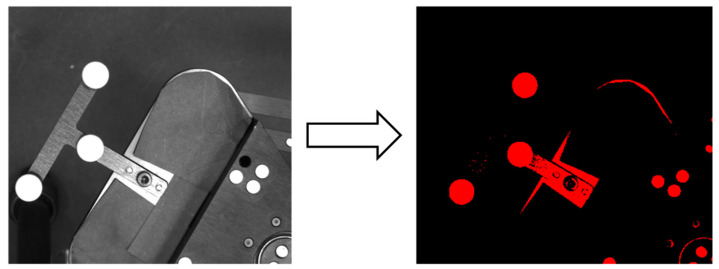
Image threshold in LabVIEW. (**left**) Grayscale image; (**right**) binary image.

Through the camera calibration, the aforementioned errors are calibrated, increasing the measurement and calculation accuracy in the machine-vision process. There are many methods for camera calibration [28,29,30,31,32,33], and in this study, calibration was performed using the camera calibration tool Vision Assistant provided by NI LabVIEW (Figure 11 and Figure 12) [34,35].

### 3.2. Image Acquisition and Processing

The vision sensor must undergo several processing steps for the accurate recognition of the reflective marker [36,37]. First, the grayscale image is converted into a binary image. The purpose of this conversion is to convert the image with grayscale data in the range of 0–255 into an image consisting of only 0 s and 1 s. This reduces the total capacity, and only reflective markers are displayed on the binary image. In LabVIEW, this process is performed using the threshold function. When the brightness of the image is expressed on the scale 0–255, the numbers from 0 to 170 correspond to 0 (black), and the numbers from 171 to 255 correspond to 1 (red) (Figure 12). 

Second, all the points in the image that have a circular shape are identified through the “Finding circle” function. At this time, as shown in Figure 12, the head of the fastening screw also has a circular shape, and these are also recognized as circles. 

Therefore, when the circle diameter is limited to 12–13 mm (so that only the reflective marker with the diameter of 12.66 mm can be recognized), only three circles are recognized, the center coordinates of these circles are acquired, and the process continues to the marker-identification step [38].

**Figure 13:**
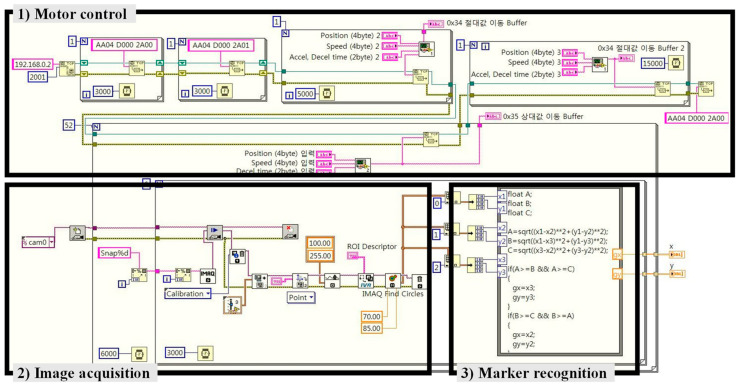
LabVIEW code for the look-then-move process.

**Figure 14:**
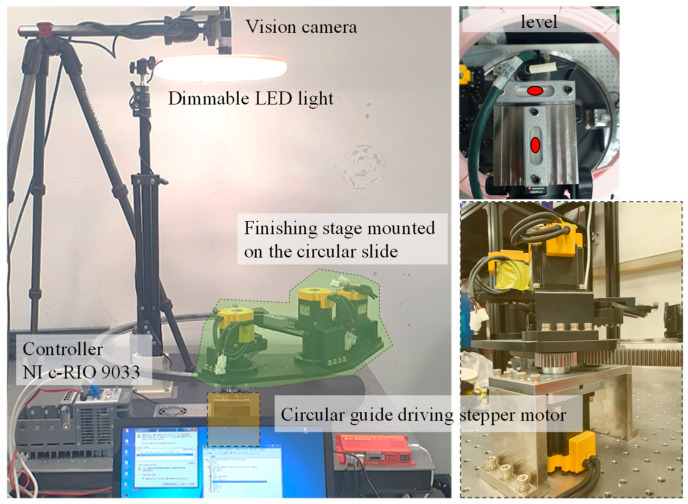
Snapshot of the experimental environment. (**left**) Side view; (**right**) top view of the vision-camera part with two levels.

Figure 13 and Figure 14 show the LabVIEW code for the machine-vision process and the hardware configuration for the experiment. After the look-then-move process, the center coordinates of the target marker at each angle were obtained through the machine-vision process, and among the circle fitting functions [39,40,41,42], the Pratt method [42] is used to estimate the origin of the frame {E} of the CMS with reference to the coordinate system {C}.

**Figure 15:**
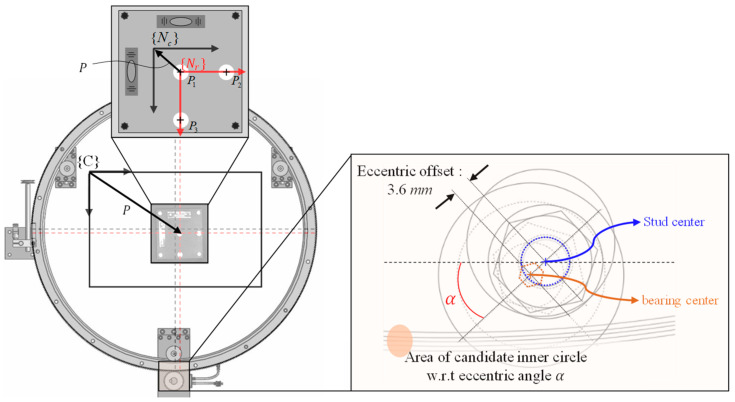
Range of the center point of the CMS with the variation of the eccentric angle.

**Figure 16:**
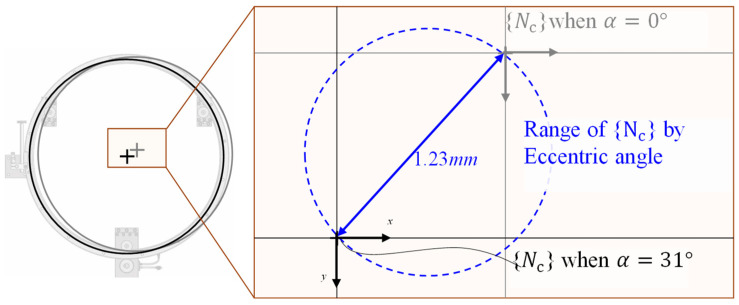
Schematic of the reference square and eccentric bearing.

The estimated origin {E} of the circular slide is compared with the nominal origin, and the following procedure is performed using the machine-vision process and design drawings to define this nominal origin. First, as shown in Figure 15, the center coordinates of the three reflective markers attached to the reference square are obtained through the machine-vision process and defined as $P_{1},\;\;P_{2}$ and $P_{3}$. The nominal reference frame {$N_{r}$} is defined by these three coordinates, and the rotation by 1.3° with reference to frame {C} is calculated. The rotation matrix RNrC and PNrC at this point are presented in Table 3 and Equation (8). Second, the nominal center frame {$N_{c}$} of the CMS is defined in the design drawing. In this case, the nominal origin is the center of the circle with the V-surface edge line of the CMS to be connected to the aforementioned three V bearings as the circumference. Because it is fixed by the eccentric bearing, to calculate {$N_{c}$}, the center coordinates obtained with all three bearings set as the center and the diameter of the center coordinate trajectory of the CMS obtained with reference to the eccentric bearing offset of 3.6 mm. Therefore, owing to the distance error from the origin of the frame {E}, the error rate occurs in this range. In the design, it is determined that the fastening angle α of the eccentric bearing is between 0° and 31°, and the resulting diameter 1.23 mm of the center coordinate trajectory of the CMS is shown in Figure 15. Additionally, in Figure 16, the V-surface edged lines that can be connected for each angle α are shown.

(9) RNrC=[0.99−0.02300.0230.990001]

(10) PNrC+RNrC⋅PNcNr=PNcC

Therefore, the nominal origin coordinate PNcC can be obtained from the estimated {$N_{r}$} with reference to the coordinate system {C} and the already known PNcNr, as follows: 

### 3.3. Analysis of Experimental Results

An experiment was performed in which the light intensity and the angle shifting interval of the look-then-move process were varied. The conditions were 480 and 770 lux and 5°, 10°, and 20°, respectively. Table 4 and Table 5 present the mean value and the standard deviation (STD) of the estimated radius for each condition, as well as the distance error, which was the in-plane alignment error.

**Figure 17:**
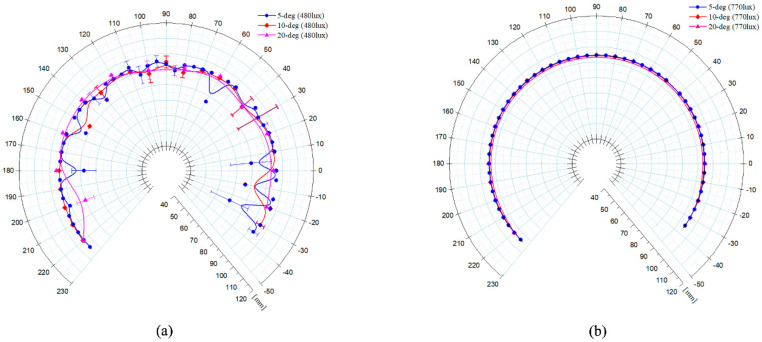
Results of circle fittings with respect to the frame {C} for different angle shifting intervals and LED light intensities: (**a**) 480 lux; (**b**) 770 lux.

As indicated by the experimental results, the distance error varied by up to 640%, depending on the light intensity. A circle fitted at 770 lux exhibited little variation with regard to the radii estimated at all the angles and the STD, whereas a circle fitted at 480 lux exhibited a large variation. This is a problem, as the vision sensor was unable to properly recognize the target marker under the low light intensity, as confirmed by Figure 17a. Even after the deviations of the values were calibrated using the Pratt method, the results were unreliable, indicating an adverse effect on the precision.

**Figure 18:**
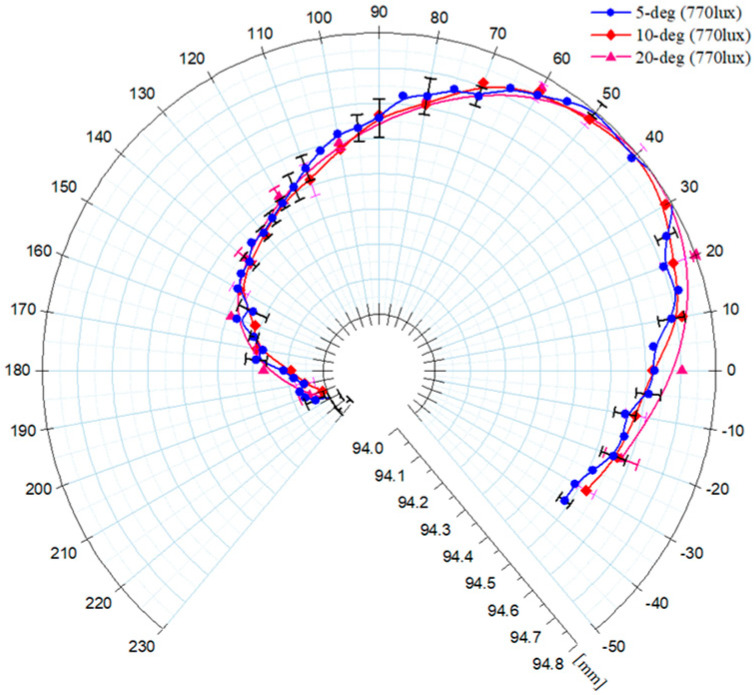
Results of circle fittings with respect to frame {Nc} under the 770-lux light-intensity condition.

Figure 18 shows the origin fitting at 770 lux, shown at a scale within 0.8 mm in total. The results were valid, as they were almost independent of the angle shifting interval. Additionally, an alignment error occurred, as evidenced by a comparison with Figure 18 with reference to the normal origin. The alignment error was 0.37 mm, and the error rate was 27% by the range of the nominal origin.

## 4. Conclusions

To calibrate the center alignment error that occurs when a CMS is used for expanding the workspace of the parallel mechanism, a method for determining the error relative to the normal origin was developed. The method involves rotating the mechanism with a reflective marker attached and defining the center of the circular trajectory of the marker as the actual origin.

For this purpose, a camera was selected, and the relationship between the length per unit pixel and the working distance h was selected as the camera height for the adequate calibration precision. Therefore, h is set at 565 mm, length per unit pixel is set at 0.07841 mm. Then, this value was implemented in an actual experimental environment setting. Additionally, to validate the experimental results, the experiments were conducted several times, while the light intensity and shifting angle were varied. As a result, the origin center alignment error was identified as 0.37 mm in 770 lux brightness conditions and in all angles.

Because the proposed calibration method involves a camera that can be easily installed and leveled through a tripod, it can be applied as long as the reflective-marker recognition is possible, even if the entire space is limited. Thus, it is very useful in the application of a CMS.

## Figures and Tables

**Table 1:** Information about all the hardware and software used in this study.

-	Item	Model Number	Specifications	
**Driving part**	Stepper motor	EZi-servo M2 60M	Drive method	BI-POLAR
			Pulse/revolution	20,000
			Current per PHASE	4 A
	Controller	NI Compact RIO 9033	Software	Labview
**Driven part**	Circular slide	Hepcomotion RIM 627R360 P N	Pitch diameter	606.5 mm
			Number of teeth	500
	Pinion	P125W14T34B	Number of teeth	34
**Measurement**	Vision camera	Basler acA3800-10gm	Table 2	
	Lens	TC1214-3MPG		
	Reflective marker	Optitrack MKR127M4-10	diameter	12.7 mm

**Table 2:** Specifications of the machine-vision camera and lens. FOV, field of view.

**Sensor size *n* × *t***	6.4 × 4.6 [mm]
**Focal length *f***	12 [mm]
**Camera resolution**	3840 × 2748 [pixel]
**Length per unit pixel**	0.07841 [mm]
**FOV size *w* × *d***	300 × 218 [mm]
**Working distance *h***	565 [mm]

**Table 3:** Estimated displacement vectors (mm).

Δ*P*	Δ*x*	Δ*y*
PNrC	144.90	112.60
PNcNr	0.83	−8.53
PNcC	145.73	104.07

**Table 4:** Experimental results were obtained at 480 lux (mm).

Δθ	Radius	STD	Dist. Error
20°	92.29	4.67	2.38
10°	92.74	4.35	1.98
5°	91.96	5.92	1.36

**Table 5:** Experimental results were obtained at 770 lux (mm).

Δθ	Radius	STD	Dist. Error	Error Rate
20°	94.50	0.25	0.37	27%
10°	94.46	0.25	0.37	27%
5°	94.44	0.26	0.36	27%
